# The Relationships between the Working Fluids, Process Characteristics and Products from the Modified Coaxial Electrospinning of Zein

**DOI:** 10.3390/polym11081287

**Published:** 2019-08-01

**Authors:** Menglong Wang, Tao Hai, Zhangbin Feng, Deng-Guang Yu, Yaoyao Yang, SW Annie Bligh

**Affiliations:** 1 School of Materials Science & Engineering, University of Shanghai for Science & Technology, Shanghai 200093, China; 2 Caritas Institute of Higher Education, 2 Chui Ling Lane, Tseung Kwan O, New Territories, Hong Kong 999077, China

**Keywords:** coaxial electrospinning, length of straight fluid jet, spreading angle, nanoribbons, linear relationship

## Abstract

The accurate prediction and manipulation of nanoscale product sizes is a major challenge in material processing. In this investigation, two process characteristics were explored during the modified coaxial electrospinning of zein, with the aim of understanding how this impacts the products formed. The characteristics studied were the spreading angle at the unstable region (*θ*) and the length of the straight fluid jet (*L*). An electrospinnable zein core solution was prepared and processed with a sheath comprising ethanolic solutions of LiCl. The width of the zein nanoribbons formed (*W*) was found to be more closely correlated with the spreading angle and straight fluid jet length than with the experimental parameters (the electrolyte concentrations and conductivity of the shell fluids). Linear equations *W* = 546.44*L* − 666.04 and *W* = 2255.3*θ* − 22.7 could be developed with correlation coefficients of *R*_wl_^2^ = 0.9845 and *R*_wθ_^2^ = 0.9924, respectively. These highly linear relationships reveal that the process characteristics can be very useful tools for both predicting the quality of the electrospun products, and manipulating their sizes for functional applications. This arises because any changes in the experimental parameters would have an influence on both the process characteristics and the solid products’ properties.

## 1. Introduction

For polymer processing engineering, a key requirement is to be able to accurately interrelate the experimental conditions and the properties of the final products [1,2,3]. This is particularly challenging when the final products are nanoparticles or nanofibers [4,5,6,7,8,9]. Both of them can be generated by electrohydrodynamic atomization (EHDA) using electrostatic energy [10,11,12,13,14,15,16], and while there are numerous reports of such fabrication processes, it remains the case that it is extremely difficult to predict the outcome of a given experiment.

**Figure 1:**
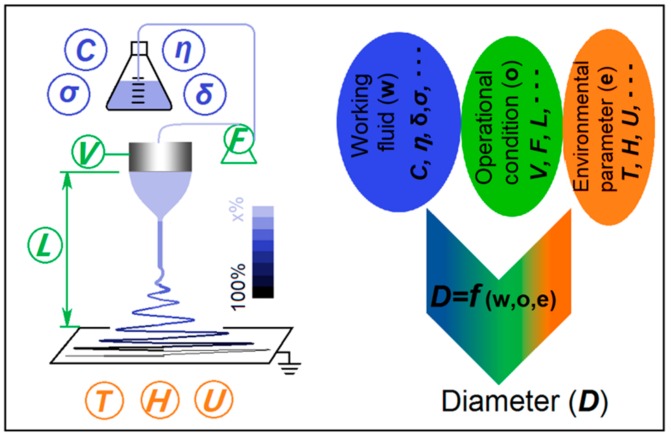
A diagram showing the single-fluid electrospinning process and the experimental parameters exerting influence on the diameters of the polymer nanofibers generated.

Figure 1 presents a schematic depicting the experimental parameters that exert a significant influence on the final polymer nanofibers generated in the simplest electrospinning experiment, which involves a single working fluid. In electrospinning, the working fluid and electrostatic energy are brought together at the nozzle of the spinneret [17,18,19,20,21]. After exiting the spinneret, the working fluid is forced to bend and whip, and during this process it is solidified into nanofibers at an extremely rapid speed [22,23,24,25,26,27]. Based on this working procedure, the key experimental parameters can be divided into three categories (see Figure 1). Correspondingly, the resultant nanofiber diameter (*D*) is a function of the working fluid properties (*w*), the operational conditions (*o*), and the environmental parameters (*e*): i.e., *D* = *f* (*w*,*o*,*e*).

Over the past two decades, electrospinning has developed very rapidly, with potential applications of polymer nanofibers having been proposed in a myriad of scientific fields [28,29,30,31]. In addition, the simple single-fluid electrospinning process has advanced to two-fluid coaxial and side-by-side processes, and tri-fluid coaxial and combined coaxial/side-by-side processes. It has proven to be possible to perform the electrospinning process even when one or more of the working fluids cannot on its own be processed: For instance, in modified coaxial electrospinning, a spinnable core solution is partnered with an unspinnable sheath fluid. These novel processes permit the production of nanofibers with increasingly complicated nanostructures [32,33,34,35,36,37]. As a result, in the literature, there are numerous publications that explore the influence of a single parameter on the nanofibers or nanostructures prepared by electrospinning, elucidating relationships for manipulating the products (mainly in terms of diameter, but also for other properties such as morphology, surface smoothness and the details of the nanostructure) [38,39,40,41,42,43]. However, there are a number of experimental parameters that can simultaneously exert an influence on the final products [44,45,46,47]. For example, the properties of working fluid include polymer concentration (*C*), viscosity (*η*), surface tension (*δ*), and conductivity (*σ*); the operational conditions include the applied voltage (*V*), the fluid flow rate (*F*), and the fiber collection distance (*L*); and the environmental parameters comprise of temperature (*T*), humidity (*H*) and the possible vacuum (*U*) (Figure 1). 

Thus, although a lot of effort has been expended to predict and manipulate the diameters of electrospun nanofibers, the results are typically far from satisfactory [48,49]. During electrospinning, almost all the experimental parameters can influence the working process, and furthermore, they are not independent variables, and can also influence each other. For example, the flow rate of the working fluid and the applied voltage need to be matched, or droplets of working fluid may fall directly onto the fiber collector. Thus, although many mathematical models have been put forward for a particular working fluid [48], they often fail to be applicable to other situations. Although the experimental parameters have drawn intensive attention, it is strange that the detailed steps in the process of electrospinning have received very limited attention. These include the formation of the Taylor cone, the ejection of a straight fluid jet, and also the bending and whipping region [50,51]. These individual steps are influenced by all the experimental parameters, and thus can be directly controlled by researchers. It is hypothesized that the nature of each of these stages of the spinning process should have a distinct relationship with the final nanofiber properties, particularly their sizes. 

Here, for the first time, we design a method to elucidate the interrelated relationships between the working fluids, the electrospinning process characteristics, and the nanoribbons formed during the modified coaxial electrospinning of zein. Zein is one of the best understood plant proteins. Extracted from maize, it has been widely used as a coating for candy, nuts, fruit, and pharmaceuticals. Zein can be processed into resins and other bioplastic polymers, which can be extruded or rolled into a variety of plastic products [52]. Zein has good processability using both these traditional technologies and advanced technologies such as electrospinning and electrospraying [53,54,55]. Here, it was selected as a model system for a detailed exploration of the individual stages in the electrospinning process. A series of modified coaxial electrospinning processes were carried out and several types of zein nanoribbons were fabricated. The working processes were digitally recorded and quantitatively described in terms of length of the straight fluid jet and the spreading angle of the unstable region, and these were interrelated with both the initial conductivity and the final zein nanoribbon widths. 

## 2. Materials and Methods 

### 2.1. Materials 

Zein (98% purity) was purchased from Shanghai Hewu Biotechnology Co., Ltd. (Shanghai, China). Anhydrous ethanol and lithium chloride were obtained from the Husheng Reagent Co., Ltd. (Shanghai, China). Water was double distilled just before use.

### 2.2. Modified Coaxial Electrospraying

The core fluid consisted of 28 g zein in 100 mL of a 75%/25% (*v*/*v*) ethanol/water mixture, which showed a yellow color. Four LiCl solutions in ethanol (at 0, 5, 10, and 20 mg/mL) were utilized as the sheath fluids, and the resultant nanoribbons were labeled as Z1, Z2, Z3, and Z4, respectively. The conductivities of the sheath fluids were assessed using a DDS-11 digital conductivity meter (Shanghai Rex Co-perfect Instrument Co., Ltd., Shanghai, China).

A homemade system was employed to conduct all the electrospinning processes. This consisted of two syringe pumps (KDS100 and KDS200, Cole–Parmer, Vernon Hills, IL, USA), a power supply (ZGF200, 60 kV/2 mA, Wuhan Huatian Corp., Wuhan, China), a homemade concentric spinneret, and an aluminum-coated flat piece of cardboard as the collector. The ambient temperature and humidity were 21 ± 4 °C and 53 ± 6%, respectively. All the working processes were recorded using a digital camera (PowerShot A490; Canon, Tokyo, Japan). Following optimization, the collection distance and voltage were fixed at 20 cm and 17 kV, respectively.

### 2.3. Morphology of the Prepared Nanoparticles

The surface morphologies of the electrospun products were observed by scanning electron microscopy (SEM; Quanta FEG450, FEI Corporation, Hillsboro, OR, USA) at 20 kV acceleration voltage. Before observation, the samples were sputter-coated with gold under vacuum. The images were analyzed using the ImageJ software (National Institutes of Health, Bethesda, MD, USA), with measurements taken at over 100 different places to determine the average ribbon diameter.

## 3. Results and Discussion

### 3.1. Implementation of Modified Coaxial Electrospinning

**Figure 2:**
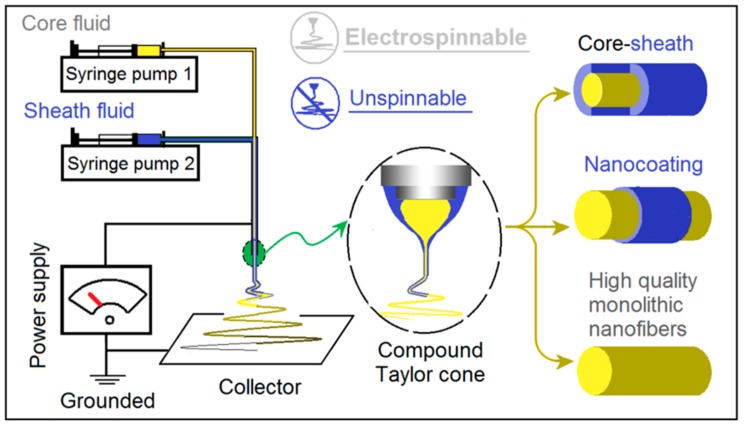
The modified coaxial electrospinning process, which permits a range of novel structures to be obtained through the unspinnable sheath fluid.

Traditionally, coaxial electrospinning is carried out using an electrospinnable sheath fluid to encapsulate either a core liquid which may be spinnable or unspinnable [18,24]. Some years ago, Yu and co-workers expanded this concept to develop the modified coaxial process, with an unspinnable liquid as the sheath fluid (Figure 2). 

**Figure 3:**
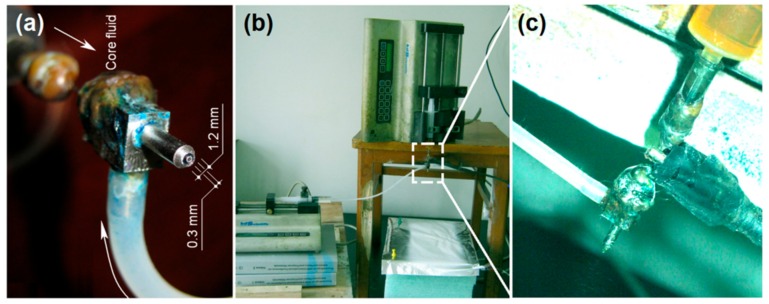
The apparatus used for modified coaxial electrospinning: (**a**) The home-made concentration spinneret; (**b**) the arrangement of apparatus; and (**c**) the connection of the power supply and working fluids with the spinneret.

The homemade concentric spinneret and the electrospinning apparatus used in this work are shown in Figure 3. The spinneret (Figure 3a) consists of a narrow metal capillary (inner diameter 0.3 mm, wall thickness 0.1 mm) nested into an outer capillary (inner diameter of 1.2 mm, wall thickness 0.2 mm). Two syringe pumps were employed to drive the core and shell liquids to the spinneret (Figure 3b). The yellow zein solution was directly guided to the inner needle of the spinneret through a plastic syringe, whereas the sheath LiCl solution was pumped to the spinneret through elastic silicon tubing. An alligator clip connects the spinneret to the power supply and carries electrostatic energy to the working fluid (Figure 3c). 

### 3.2. The Working Processes and the Resultant Zein Nanoribbons

**Figure 4:**
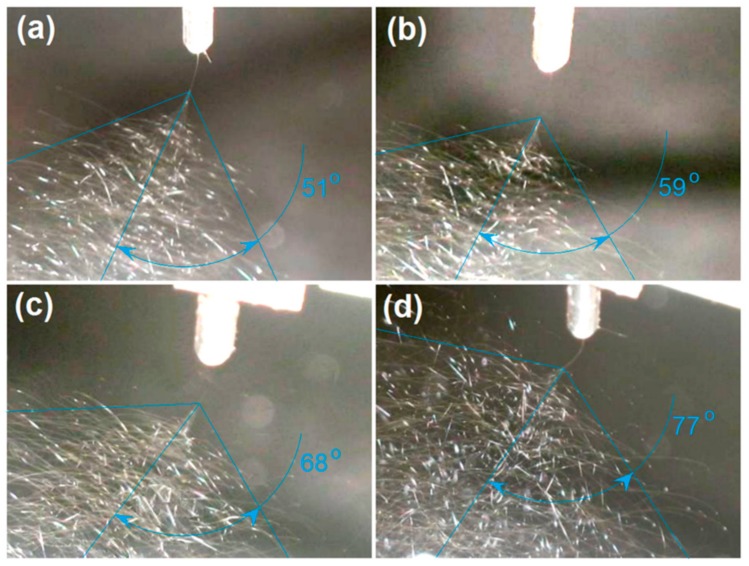
The changes of spreading angle and the length of straight fluid jet with the increase of LiCl in the sheath solution (mg/mL): (**a**) 0; (**b**) 5; (**c**) 10; (**d**) 20.

The electrospinning process consists of three successive stages: The formation of a Taylor cone, the straight fluid jet emitted from the Taylor cone, and the unstable region, which is composed of numerous bending and whipping loops. The formation of the Taylor cone is a balance between the electrical force exerted on the droplets exiting the spinneret, and the surface tension of the working fluids. When the conductivity of the working fluid increases, the electrical forces should increase correspondingly. Thus, an increase in the LiCl concentration in the sheath fluid is expected to result in a stronger electrical force being applied to the working fluids. Under the same applied voltage and spinneret-to-collector distance, this force will greatly change the behaviors of the working fluids. Digital photographs of these are given in Figure 4. As the LiCl concentration increased from 0 to 5, and from 10 to 20 mg/mL, the length of the straight fluid jet decreased from 3.3 ± 0.4, to 2.9 ± 0.3, and from 2.4 ± 0.3 to 2.2 ± 0.2 mm, respectively. Meanwhile, the spreading angles of the unstable region increased from 51 ± 4° to 59 ± 5°, and from 68 ± 4° to 77 ± 6°. 

**Figure 5:**
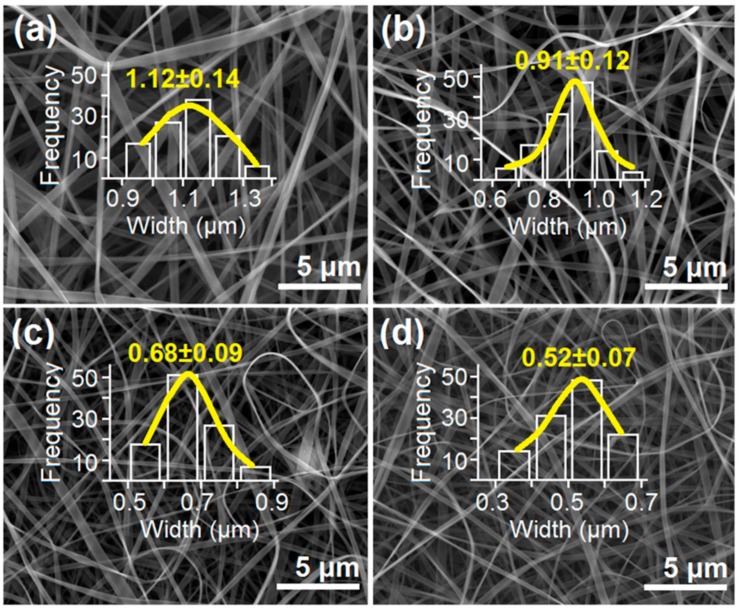
SEM images of resultant zein nanoribbons, with their width distributions. (**a**) Z1; (**b**) Z2; (**c**) Z3; (**d**) Z4.

SEM images of the resultant nanoribbons and their diameter distributions are shown in Figure 5. All the ribbons have a linear morphology. No beads or spindles are found in the ribbons, suggesting the core zein solution has good electrospinnability. Nanoribbons Z1, Z2, Z3, and Z4 have an estimated width of 1.12 ± 0.14, 0.91 ± 0.12, 0.58 ± 0.09, and 0.52 ± 0.07 μm, respectively. 

### 3.3. The Influence of Conductivity on the Behavior of the Working Fluids

Although a single-step and straightforward process for creating nanoribbons, the electrospinning process is in fact very complicated. This complexity is reflected in two ways. First, the process involves the overlap of multiple disciplines such as hydrodynamic science, polymer science and rheology, and electric dynamics. Second, a small change in the working fluid properties can greatly influence the process and its products. 

**Figure 6:**
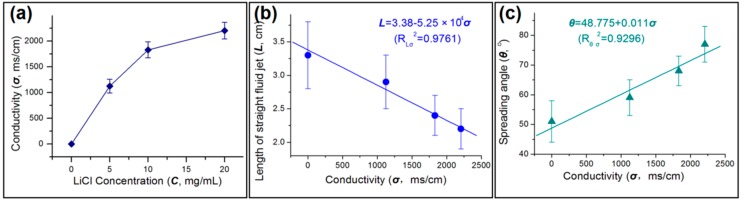
The influence of the sheath fluid conductivity on the behavior of the working fluids: (**a**) The relationship between LiCl concentration and solution conductivity; (**b**) the decrease in the length of the straight fluid jet with an increase of conductivity; (**c**) the increase of spreading angle with rising conductivity.

As the concentration of LiCl increased, the conductivity of the sheath solution also rose (Figure 6a). This increase in conductivity will make the solution subject to stronger electrical forces, which in turn alter the behavior of both the sheath and core working fluids. The length of the straight fluid jets gradually decreased with conductivity in a linear fashion, as shown in Figure 6b: *L* = 3.38 − 5.25 × 10^4^ *σ,* with a correlation coefficient of *R*_Lσ_^2^ = 0.9761. Similarly, the spreading angle of the unstable region gradually increased with conductivity (Figure 6c). A linear equation *θ* = 48.775 + 0.011 *σ* can be fitted to the data, giving a correlation coefficient of *R*_θσ_^2^ = 0.9296. The clearly linear nature of the plots in Figure 6b, c and the high *R*^2^ values obtained show there are clear causal relationships here. 

In literature, numerous publications have investigated the electrospinnability of a certain working fluid, which is mainly determined by the type of filament-forming polymer, its concentrations in the working fluid, and the applied voltage. After the past two decades’ effort, near 200 polymers can be processed into fibers using electrospinning. However, few efforts have been focused on the behaviors of working fluids within their electrospinnable windows. Knowledge about the adaptability of working fluids under the high electrical field should be useful for manipulating the fluid processing process in a more intentional manner. 

### 3.4. The Effect of Sheath Working Fluid Properties on the Width of Zein Nanoribbons 

**Figure 7:**
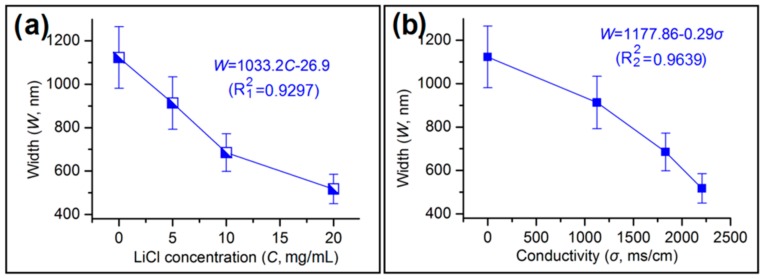
Correlations between the width of electrospun zein nanoribbons with: (**a**) The LiCl concentration; and (**b**) the conductivity of the sheath fluid.

A wide variety of experimental parameters have been investigated in terms of their effect on the properties of electrospun fibers, and the solution conductivity of working fluid is recognized as being of major importance [56]. In this study, the electrolyte LiCl was added only into the sheath working fluid, because charges are always concentrated on the surface of the Taylor cone. The width of the zein ribbons produced is clearly correlated with the LiCl concentration in the sheath fluid, with a good fit to the data obtained with the linear equation *W* = 1033.2*C* − 26.9 (*R*_1_^2^ = 0.9297; Figure 7a). A similar linear equation is observed when plotting ribbon width as a function of sheath solution conductivity (*W* = 11177.86 − 0.29*σ*; *R*_2_^2^ = 0.9639). These linear equations suggested that the LiCl concentration and conductivity of sheath working fluid directly influenced the width of the zein nanoribbons fabricated. These equations can hence be exploited to predict the size of the products from this electrospinning process, and provide useful information for optimizing the working processes. 

It is a common strategy to optimize the experimental conditions through simultaneous investigations on several levels of an experimental parameter, just as here with the LiCl concentration. However, only a small part of the related publications has taken a further step to disclose the inherent relationship between the vital properties of the working fluid with the final product’s quality. Here, the conductivity of sheath LiCl solution showed a better linear relationship with the width of zein ribbons than the LiCl concentration. Thus, among many other solution properties such as surface tension, viscosity, and rheological properties, conductivity is the most important property of LiCl solution that exerted influences on both the working processes, and also the resultant ribbons’ quality. 

### 3.5. Correlations between the Width of Electrospun Zein Nanoribbons and the Detailed Steps of Electrospinning 

**Figure 8:**
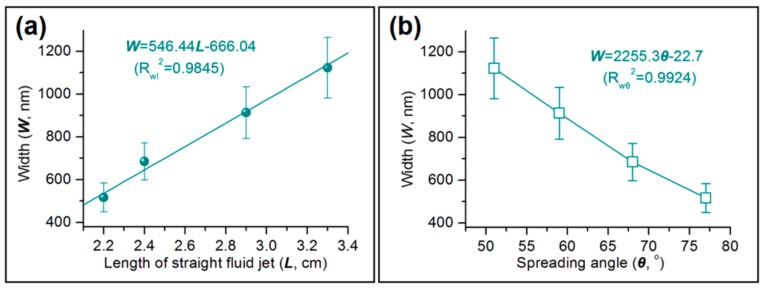
The correlations between the width of electrospun zein nanoribbons and: (**a**) The length of the straight fluid jet; and (**b**) the spreading angle of the unstable zone.

The detailed observations of the electrospinning process discussed in Section 3.2 have a very close relationship with the size of the zein ribbons produced (Figure 8). A linear equation relates the width of the ribbons to the length of the straight fluid jet (*W* = 546.44*L* − 666.04; *R*_wl_^2^ = 0.9845). Similarly, a linear equation *W* = 2255.3θ − 22.7 connects the width of the ribbons with the spreading angle (*R*_wθ_^2^ = 0.9924). These relationships show that these parameters can be very useful tools for predicting the properties of the ribbons fabricated. 

Right from the rebirth of electrospinning, a wide variety of experimental parameters have been studied to disclose their potential roles during the electrospinning processes. These parameters can all be manipulated by the researchers directly and changed within a certain range, which are concluded in Figure 1. However, these parameters often result in interrelated influences. For example, an increase of LiCl concentration resulted in a larger conductivity, but also changed the working fluid’s surface tension, viscosity, and exerted on the effect of applied voltage. Thus, although many publications have reported the relationships between a certain experimental parameter and the final nanoproducts’ size. It is difficult to disclose their relationship in an accurate manner. In contrast, the process characteristics, similarly as the final product to be influenced systematically from all the experimental parameters, have the essential advantages over the processing parameters in predicting the final nanoproducts’ size, and in providing useful information for accurate and robust manipulation of the processing process. 

### 3.6. The Role of Process Characteristics

**Figure 9:**
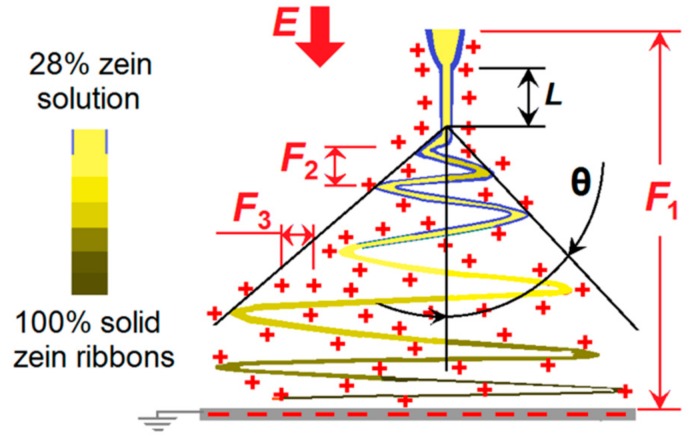
A diagram showing the formation mechanism of electrospun nanoribbons through the modified coaxial electrospinning.

A schematic diagram of the modified coaxial electrospinning process is presented in Figure 9. Initially, the sheath LiCl solution surrounds the core zein solution to form a compound Taylor cone. The two fluids come through the straight fluid jet and enter the unstable region together. During the early stages of the unstable region, the sheath solution will be evaporated, and then later, the core zein solution will be gradually dried during the drawing processes. A series of different forces will be exerted on the working fluids, such as the force between the two electrodes (*F*_1_) and gravity (*G,* which can often be neglected). Within the bending and looping fluid jets, repulsive forces will include those between different loops (*F*_2_) and those within the different parts of a single loop (*F*_3_). It is the *F*_3_ forces that draw and narrow the working fluids. The spreading angle will be a parameter that reflects the combined actions of *F*_1_, *F*_2_, and *F*_3_. An increase in sheath solution conductivity will increase all three forces. An increase in *F*_1_ would act to decrease the spreading angle. However, an increase in *F*_2_ would make the fluid travel time increase during the drawing process, and thus provide a trend of enlarging the spreading angle. In addition, an increase in *F*_3_ would make the loops larger, and correspondingly increase the spreading angle. Thus, the combined effects of *F*_2_ and *F*_3_ appear to have a more marked influence on the electrospinning process than *F*_1_, and as a result, the greater the conductivity of the sheath fluid, the larger the spreading angle observed. Similarly, another process characteristic, i.e., the length of straight fluid jet, has received the influences of LiCl concentration directly and comprehensively.

In the biomedical applications of electrospun nanofibers, whether for tissue engineering or advanced drug delivery systems, the accurate manipulation of nanofiber diameter is very important for the fibers’ functional performances [57,58,59]. This work reveals that the process characteristics (the length of straight fluid jet and the spreading angle of unstable region) have a close linear relationship with the final nanoribbon width, and can provide useful information for manipulating the working processes, and developing products with the desired physical properties. 

## 4. Conclusions and Perspectives

Using an electrospinnable zein solution as the core fluid and LiCl solutions as the sheath working liquids, a series of modified coaxial electrospinning processes were performed, and a number of zein nanoribbons successfully prepared. The nanoribbon width (*W*) was found to be directly correlated with the concentration of LiCl (*C*) and the conductivity of the sheath fluid (*σ*), with linear relationships of the form *W* = 1033.2*C* − 26.9 (*R*_1_^2^ = 0.9297), and *W* = 11177.86 − 0.29*σ* (*R*_2_^2^ = 0.9639) determined. Further, the width of the zein nanoribbons (*W*) were found to have still closer linear relationships with the spreading angle in the unstable region (*θ*), and the length of the straight fluid jet (*L*) (*W* = 546.44*L* − 666.04 and *W* = 2255.3*θ* − 22.7; *R*_wl_^2^ = 0.9845 and *R*_wθ_^2^ = 0.9924, respectively).

Today, electrospun nanofibers are rapidly approaching commercial applications in several fields such as drug delivery, food packaging, water treatment, and air filtration [60,61], and the production of electrospun nanofibers on a large scale is now possible [62]. Two important issues will require attention for accelerating nanofiber-based commodities to the market. One is the accurate and robust control of the processing process during electrospinning. The second is the prediction and maintenance of the nanofiber quality. For resolving these two issues, the characteristics of the working process itself offer a powerful source of information, and have advantages over the processing parameters (i.e., those that can be manipulated directly by the operator). This is because these working process characteristics manifest the simultaneous influence of all the processing parameters, as do the fibers produced. Thus, it is anticipated that they can act as a useful tool for stabilizing the working process, for systematic manipulation of the processing parameters, and for accurately predicting the resultant nanofiber size. Similar observations have been noted for electrospraying, an alternative electrohydrodynamic atomization process [63]. 

## References

[1] Dotivala A.C., Puthuveetil K.P., Tang C. (2019). Shear force fiber spinning: Process parameter and polymer solution property considerations. Polymers.

[2] Vass P., Démuth B., Hirsch E., Nagy B., Andersen S.K., Vigh T., Verreck G., Csontos I., Nagy Z.K., Marosi G. (2019). Drying technology strategies for colontargeted oral delivery of biopharmaceuticals. J. Control. Release.

[3] Wu H., Zhao S., Ding W., Han L. (2018). Studies of interfacial interaction between polymer components on helical nanofiber formation via coelectrospinning. Polymers.

[4] Vicente A.C.B., Medeiros G.B., do Carmo Vieira D., Garcia F.P., Nakamura C.V., Muniz E.C., Corradini E. (2019). Influence of process variables on the yield and diameter of zeinpoly (n-isopropylacrylamide) fiber blends obtained by electrospinning. J. Mol. Liquids.

[5] Acik G., Cansoy C.E., Kamaci M. (2019). Effect of flow rate on wetting and optical properties of electrospun poly (vinyl acetate) microfibers. Colloid Polym. Sci..

[6] Liu M., Zhang Y., Sun S., Khan A.R., Ji J., Yang M., Zhai G. (2019). Recent advances in electrospun for drug delivery purpose. J. Drug Target..

[7] Chakrabarty A., Teramoto Y. (2018). Recent advances in nanocellulose composites with polymers: A guide for choosing partners and how to incorporate them. Polymers.

[8] Haider A., Haider S., Kang I.K. (2018). A comprehensive review summarizing the effect of electrospinning parameters and potential applications of nanofibers in biomedical and biotechnology. Arab. J. Chem..

[9] Okutan N., Terzi P., Altay F. (2014). Affecting parameters on electrospinning process and characterization of electrospun gelatin nanofibers. Food Hydrocolloids.

[10] Chen B.Y., Lung Y.C., Kuo C.C., Liang F.C., Tsai T.L., Jiang D.H., Satoh T., Jeng R.J. (2018). Novel multifunctional luminescent electrospun fluorescent nanofiber chemosensorfilters and their versatile sensing of pH, temperature, and metal ions. Polymers.

[11] Jiang D.H., Tsai P.C., Kuo C.C., Jhuang F.C., Guo H.C., Chen S.P., Tung S.H. (2019). Facile preparation of Cu/Ag Core/Shell electrospun nanofibers as highly stable and flexible transparent conductive electrodes for optoelectronic devices. ACS Appl. Mat. Interfaces.

[12] Yu D.G., Zheng X.L., Yang Y., Li X.Y., Williams G.R., Zhao M. (2019). Immediate release of helicid from nanoparticles produced by modified coaxial electrospraying. Appl. Surf. Sci..

[13] Li X.Y., Zheng Z.B., Yu D.G., Liu X.K., Qu Y.L., Li H.L. (2017). Electrosprayed sperical ethylcellulose nanoparticles for an improved sustainedrelease profile of anticancer drug. Cellulose.

[14] Wang K., Wen H.F., Yu D.G., Yang Y., Zhang D.F. (2018). Electrosprayed hydrophilic nanocomposites coated with shellac for colonspecific delayed drug delivery. Mat. Design.

[15] Liu Z.P., Zhang L.L., Yang Y.Y., Wu D., Jiang G., Yu D.G. (2018). Preparing composite nanoparticles for immediate drug release by modifying electrohydrodynamic interfaces during electrospraying. Powder Technol..

[16] Yew C., Azari P., Choi J., Muhamad F., PingguanMurphy B. (2018). Electrospun polycaprolactone nanofibers as a reaction membrane for lateral flow assay. Polymers.

[17] Kijeńska E., Swieszkowski W. (2017). General requirements of electrospun materials for tissue engineering: Setups and strategy for successful electrospinning in laboratory and industry. Electrospun Mat. Tissue Eng. Biomed. Appl..

[18] Zhou H., Shi Z., Wan X., Fang H., Yu D.G., Chen X., Liu P. (2019). The relationships between the process parameters and the polymeric nanofibers fabricated using a modified coaxial electrospinning. Nanomaterials.

[19] Chlanda A., Kijeńska E., Rinoldi C., Tarnowski M., Wierzchoń T., Swieszkowski W. (2018). Structure and physicomechanical properties of low temperature plasma treated electrospun nanofibrous scaffolds examined with atomic force microscopy. Micron.

[20] Wu Y.H., Li H.P., Shi X.X., Wan J., Liu Y.F., Yu D.G. (2016). Effective utilization of the electrostatic repulsion for improved alignment of electrospun nanofibers. J. Nanomat..

[21] Yao C.H., Yang S.P., Chen Y.S., Chen K.Y. (2019). Electrospun poly (γ–glutamic acid)/β–tricalcium phosphate composite fibrous mats for bone regeneration. Polymers.

[22] Liu Y., Liang X., Wang S., Qin W., Zhang Q. (2018). Electrospun antimicrobial polylactic acid/tea polyphenol nanofibers for foodpackaging applications. Polymers.

[23] Caimi S., Wu H., Morbidelli M. (2018). PVdF-HFP and ionic-liquid-based, freestanding thin separator for lithium-ion batteries. ACS Appl. Energy Mat..

[24] Yu D.G., Li J.J., Williams G.R., Zhao M. (2018). Electrospun amorphous solid dispersions of poorly watersoluble drugs: A review. J. Control. Release.

[25] Jin M., Yu D.G., Wang X., Geraldes C.F.G.C., Williams G.R., Annie Bligh S.W. (2016). Electrospun contrast agent-loaded fibers for colon-targeted MRI. Adv. Healthc. Mat..

[26] Lee H., Inoue Y., Kim M., Ren X., Kim I. (2018). Effective formation of welldefined polymeric microfibers and nanofibers with exceptional uniformity by simple mechanical needle spinning. Polymers.

[27] Domokos A., Balogh A., Dénes D., Nyerges G., Ződi L., Farkas B., Marosi G., Nagy Z.K. (2019). Continuous manufacturing of orally dissolving webs containing a poorly soluble drug via electrospinning. Euro. J. Pharma. Sci..

[28] Saghazadeh S., Rinoldi C., Schot M., Kashaf S.S., Sharifi F., Jalilian E., Nuutila K., Giatsidis G., Mostafalu P., Derakhshandeh H. (2018). Drug delivery systems and materials for wound healing applications. Adv. Drug Deliv. Rev..

[29] Yang W., Zhang M., Li X., Jiang J., Sousa A.M., Zhao Q., Pontious S., Liu L. (2019). Incorporation of tannic acid in foodgrade guar gum fibrous mats by electrospinning technique. Polymers.

[30] Zhang Y., Zhang Y., Zhu Z., Jiao X., Shang Y., Wen Y. (2019). Encapsulation of thymol in biodegradable nanofiber via coaxial eletrospinning and applications in fruit preservation. J. Agric. Food Chem..

[31] Lv S., Zhao X., Shi L., Zhang G., Wang S., Kang W., Zhuang X. (2018). Preparation and properties of SCPLA/PMMA transparent nanofiber air filter. Polymers.

[32] Liu X., Yang Y., Yu D.G., Zhu M.J., Zhao M., Williams G.R. (2019). Tunable zeroorder drug delivery systems created by modified triaxial electrospinning. Chem. Eng. J..

[33] Yang Y., Li W., Yu D.G., Wang G., Williams G.R., Zhang Z. (2019). Tunable drug release from nanofibers coated with blank cellulose acetate layers fabricated using triaxial electrospinning. Carbohydr. Polym..

[34] Guarino V., Caputo T., Calcagnile P., Altobelli R., Demitri C., Ambrosio L. (2018). Core/shell cellulose-based microspheres for oral administration of ketoprofen lysinate. J. Biomed. Mat. Res. Part B.

[35] Costantini M., Colosi C., Święszkowski W., Barbetta A. (2018). Coaxial wetspinning in 3d bioprinting: State of the art and future perspective of microfluidic integration. Biofabrication.

[36] Caimi S., Timmerer E., Banfi M., Storti G., Morbidelli M. (2018). Core-shell morphology of redispersible powders in polymer-cement waterproof mortars. Polymers.

[37] Yu D.G., Yang C., Jin M., Williams G.R., Zou H., Wang X., Bligh S.A. (2016). Medicated Janus fibers fabricated using a tefloncoated sidebyside spinneret. Colloids Surf. B.

[38] Hou J., Wang Y., Xue H., Dou Y. (2018). Biomimetic growth of hydroxyapatite on electrospun CA/PVP core–shell nanofiber membranes. Polymers.

[39] Liao Y., Loh C.H., Tian M., Wang R., Fane A.G. (2018). Progress in electrospun polymeric nanofibrous membranes for water treatment: Fabrication, modification and applications. Progr. Polym. Sci..

[40] Martins V.D., Cerqueira M.A., Fuciños P., GarridoMaestu A., Curto J.M., Pastrana L.M. (2018). Active bilayer cellulosebased films: Development and characterization. Cellulose.

[41] Naeem M., Lv P., Zhou H., Naveed T., Wei Q. (2018). A novel in situ selfassembling fabrication method for bacterial celluloseelectrospun nanofiber hybrid structures. Polymers.

[42] Yoon J., Yang H.S., Lee B.S., Yu W.R. (2018). Recent progress in coaxial electrospinning: New parameters, various structures, and wide applications. Adv. Mat..

[43] Li J., Xu S., Hassan M., Shao J., Ren L.F., He Y. (2019). Effective modeling and optimization of pvdf–ptfe electrospinning parameters and membrane distillation process by response surface methodology. J. Appl. Polym. Sci..

[44] Liu Y.Q., He C.H., Li X.X., He J.H. (2018). Fabrication of beltlike fibers by electrospinning. Polymers.

[45] Abudula T., Saeed U., Salah N., Memic A., AlTuraif H. (2018). Study of electrospinning parameters and collection methods on size distribution and orientation of PLA/PBS hybrid fiber using digital image processing. J. Nanosci. Nanotechnol..

[46] Xia H., Chen T., Hu C., Xie K. (2018). Recent advances of the polymer micro/nanofiber fluorescence wave guide. Polymers.

[47] Szabó E., Démuth B., Nagy B., Molnár K., Farkas A., Szabó B., Balogh A., Hirsch E., Marosi G., Nagy Z. (2018). Scaledup preparation of drugloaded electrospun polymer fibres and investigation of their continuous processing to tablet form. Express Polym. Lett..

[48] Rutledge G.C., Fridrikh S.V. (2007). Formation of fibers by electrospinning. Adv. Drug Deliv. Rev..

[49] Yeo L.Y., Friend J.R. (2006). Electrospinning carbon nanotube polymer composite nanofibers. J. Exp. Nanosci..

[50] Wang Q., Yu D.G., Zhang L.L., Liu X.K., Deng Y.C., Zhao M. (2017). Electrospun hypromellosebased hydrophilic composites for rapid dissolution of poorly watersoluble drug. Carbohydr. Polym..

[51] Hai T., Wan X., Yu D.G., Wang K., Yang Y., Liu Z.P. (2019). Electrospun lipidcoated medicated nanocomposites for an improved drug sustainedrelease profile. Mat. Design.

[52] Liu Z.P., Zhang Y.Y., Yu D.G., Wu D., Li H.L. (2018). Fabrication of sustained-release zein nanoparticles via modified coaxial electrospraying. Chem. Eng. J..

[53] Dehcheshmeh M.A., Fathi M. (2019). Production of coreshell nanofibers from zein and tragacanth for encapsulation of saffron extract. Int. J. Bio. Macromol..

[54] Ghalei S., Asadi H., Ghalei B. (2018). Zein nanoparticle-embedded electrospun PVA nanofibers as wound dressing for topical delivery of anti-inflammatory diclofenac. J. Appl. Polym. Sci..

[55] Deng L., Zhang X., Li Y., Que F., Kang X., Liu Y., Feng F., Zhang H. (2018). Characterization of gelatin/zein nanofibers by hybrid electrospinning. Food Hydrocolloids.

[56] Wu Y.H., Yang C., Li X.Y., Zhu J.Y., Yu D.G. (2016). Medicated nanofibers fabricated using NaCl solutions as shell fluids in a modified coaxial electrospinning. J. Nanomat..

[57] Kouhi M., Fathi M., Reddy V.J., Ramakrishna S. (2019). Bredigite reinforced electrospun nanofibers for bone tissue engineering. Mat. Today Proc..

[58] Ngadiman N., Yusof N., Idris A., Fallahiarezoudar E., Kurniawan D. (2018). Novel processing technique to produce three dimensional polyvinyl alcohol/maghemite nanofiber scaffold suitable for hard tissues. Polymers.

[59] Sill T.J., von Recum H.A. (2008). Electrospinning: Applications in drug delivery and tissue engineering. Biomaterials.

[60] Gao S., Tang G., Hua D., Xiong R., Han J., Jiang S., Zhang Q.L., Huang C. (2019). Stimuli-responsive bio-based polymeric systems and their applications. J. Mat. Chem. B.

[61] Lv D., Zhu M., Jiang Z., Jiang S., Zhang Q., Xiong R., Huang C. (2018). Green electrospun nanofibers and their application in air filtration. Macromol. Mat. Eng..

[62] Duan G., Greiner A. (2019). Air-blowing-assisted coaxial electrospinning toward high productivity of core/sheath and hollow fibers. Macromol. Mat. Eng..

[63] Huang W., Hou Y., Lu X., Gong Z., Yang Y., Lu X.J., Liu X.L., Yu D.G. (2019). The process–property–performance relationship of medicated nanoparticles prepared by modified coaxial electrospraying. Pharmaceutics.

